# Evaporation Patterns of Dextran–Poly(Ethylene Glycol) Droplets with Changes in Wettability and Compatibility

**DOI:** 10.3390/life12030373

**Published:** 2022-03-04

**Authors:** Chiho Watanabe, Miho Yanagisawa

**Affiliations:** 1 School of Integrated Arts and Sciences, Graduate School of Integrated Sciences for Life, Hiroshima University, Kagamiyama 1-7-1, Higashi-Hiroshima 739-8521, Japan; 2 Komaba Institute for Science, Graduate School of Arts and Sciences, The University of Tokyo, Komaba 3-8-1, Tokyo 153-8902, Japan; myanagisawa@g.ecc.u-tokyo.ac.jp; 3 Universal Biology Institute, Graduate School of Arts and Sciences, The University of Tokyo, Komaba 3-8-1, Tokyo 153-8902, Japan; 4 Department of Physics, Graduate School of Science, The University of Tokyo, Hongo 7-3-1, Tokyo 113-0033, Japan

**Keywords:** evaporation pattern, dextran, PEG, phase separation, droplets

## Abstract

The dextran–PEG system is one of the most famous systems exhibiting phase separation. Various phase behaviors, including the evaporation process of the dextran–PEG system, have been studied in order to understand the physicochemical mechanism of intracellular phase separation and the effect of condensation on the origin of life. However, there have been few studies in dilute regime. In this study, we focused on such regimes and analyzed the pattern formation by evaporation. The specificity of this regime is the slow onset of phase separation due to low initial concentration, and the separated phases can have contrasting wettability to the substrate as evaporation progresses. When the polymer concentration is rather low (<5 wt%), the dextran–PEG droplets form a phase-separated pattern, consisting of PEG at the center and dextran ring of multiple strings pulling from the ring. This pattern formation is explained from the difference in wettability and compatibility between dextran and PEG upon condensation. At the initial dilute stage, the dextran-rich phase with higher wettability accumulates at the contact line of the droplet to form a ring pattern, and then forms multiple domains due to density fluctuation. The less wettable PEG phase recedes and pulls the dextran domains, causing them to deform into strings. Further condensation leads to phase separation, and the condensed PEG with improved wettability stops receding and prevents a formed circular pattern. These findings suggest that evaporation patterns of polymer blend droplets can be manipulated through changes in wettability and compatibility between polymers due to condensation, thus providing the basis to explore origins of life that are unique to the process of condensate formation from dilute systems.

## 1. Introduction

Evaporation patterns and its condensation process of colloidal suspensions on a substrate have been intensively investigated as fundamental studies for industrial applications, such as ink and printing technologies, as well as daily life phenomena, such as coffee ring pattern, i.e., a circular ring found in coffee stains [1,2,3]. These patterns are determined by the local balance between the Marangoni effect and capillary force through the modification of surface tension and viscosity [3,4]. Additionally, when phase separation (or density variation) is caused by condensation, the evaporation pattern becomes more diverse. Such an evaporation pattern with phase separation is reported as a simple blood-based diagnostic method [5,6,7] as well as a medicine quality control [8].

Recently, evaporation of biomolecular droplets has attracted attention as a mechanism for easily condensing biomolecules in relation to “intracellular phase separation” [9,10,11] and “origin of life” [12,13]. For example, the phase separation of dextran and poly (ethylene glycol) (PEG), along with the evaporation process is reported to simultaneously condensate nucleic acids and enhancement of ribozyme activity [13]. In their experimental condition, using dextran (Mw. 10 k), PEG (Mw. 8 k), and their total concentration > 2 wt%, the droplet fully adheres to the solid substrate throughout the evaporation process and the droplet shrinks as the evaporation progresses. Given that it is plausible to imagine low-concentration conditions in origin of life scenarios, the evaporation process from a much lower initial concentration becomes a relevant target for investigation. This is because it is expected to begin with a fairly dilute prebiotic soup. However, evaporation patterns with phase separation have not been reported for very low concentrations of polymer droplets.

In this study, we studied the evaporation patterns of dextran (Mw. 500 k) and PEG (Mw. 6 k) aqueous solution in dilute regimes, which also shows phase separation as evaporation progresses. The difference from previous studies lies in kinetic coupling between compatibility and wettability, i.e., the slow onset of phase separation due to low initial concentration and the wettability reversal due to concentration change. At the lower concentration (<5 wt%), dextran-rich ring-like deposition occurs at the contact line of the droplet together with phase separation progression. Then, the pinning of the PEG-rich phase is released as evaporation continues to recede the contact line inward of the droplet, dragging the high viscosity dextran-rich phase from the initial contact line. This process creates multiple string-like pattern of dextran-rich phase on the substrate. This pattern is not observed for higher initial concentration solution since the receding of the contact line does not take place due to its high viscosity, nor when the overall viscosity is low, and the entire droplet moves together. This specific string pattern formation was not observed in a previous report using the dextran–PEG system [13], possibly due to their conditions using higher polymer concentrations and a lower molecular weight of dextran. These findings suggest that evaporation patterns of polymer blend droplets can be manipulated through changes in wettability and compatibility between polymers due to condensation. In addition, they will help us to develop a plausible prebiotic model as well as industrial applications, such as self-organizing patterning and body-fluid-based medical diagnostics using a little sample solution.

## 2. Materials and Methods

### 2.1. Materials

Dextran from *Leuconostoc mesenteroides*, with an average molecular weight of 500,000 (500 k), and fluorescein isothiocyanate–dextran (FITC–Dex 500 k) were purchased from Sigma-Aldrich (Saint Louis, MO, USA). PEG, with an average molecular weight 6000 (6 k), and glucose were purchased from FUJIFILM Wako Pure Chemical Corporation (Osaka, Japan). Rhodamine B-labeled PEG (RB-PEG) (molecular weight of 5000, 5 k) was purchased from Biochempeg (Watertown, MA, USA). These materials were used without further purification. Milli-Q water (18.2 MΩ cm, Merck Millipore, Meguro, Japan) was used for all solutions. Each polymer or glucose solution was prepared as a high-concentration mother solution (10–40 wt%), and then mixed and/or diluted by water to obtain desired composition and concentration. Rectangular cover slips were purchased from Matsunami Glass Inc., Ltd., Osaka, Japan. All chemicals and materials were used without further purification. The initial concentrations of fluorescent reagents were 0.05 mg/mL and 0.005 mg/mL for FITC–Dex and RB-PEG, respectively. Similarly to our previous reports [14,15], the radius of gyration *R*_g_ and overlap concentration c* of dextran 500 k and PEG 6 k are estimated and shown in Table 1.

### 2.2. Sessile Droplet Formation and Its Observation

**Figure 1:**
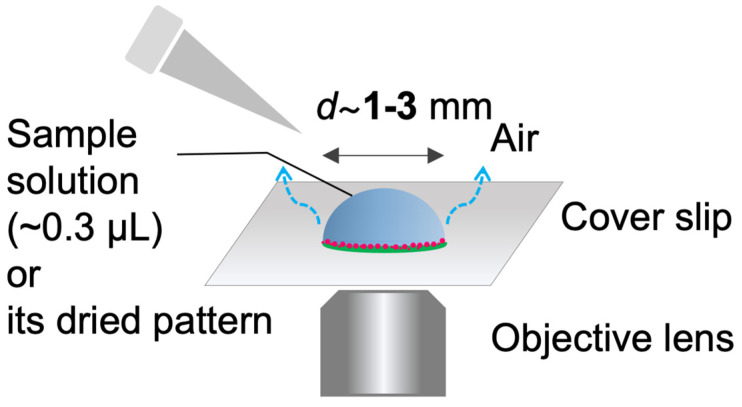
Illustration of the experimental setup.

Sessile droplets were formed with volume of ca. 0.3 µL on a cover glass and dried under ambient temperature (20–24 °C). The relative humidity was not controlled in the experiments. Typically, the relative humilities for summer and winter are 65 ± 10% and 30 ± 10%, respectively. The drying process and/or the final deposition pattern were observed by inverted fluorescence microscope (IX 73, Olympus Corp., Ltd., Tokyo, Japan). The pattern images were taken phase contrast (PH) or fluorescence (FL) by using fluorescent mirror unit (U-FBNA for FITC–Dex and U-FGW for RB-PEG). All images were taken using a color camera (DP74). The microscope, mirror units, and the camera are from Olympus Corp. Image analyses were performed by using a free-software Fiji from the National Institute of Health (USA). The experimental setup is illustrated in Figure 1. 

### 2.3. Contact Angle Measurement

The contact angle was measured by the contact angle meter SImage Entry 6 (Excimer, Inc., Kanagawa, Japan) with the *θ*/2 method. The initial contact angle was measured within 10 s after depositing ca. 2 µL solution onto the cover slip. The measurement was repeated at least 3 times to take the average value.

## 3. Results and Discussion

### 3.1. Evaporation Patterns for Dextran–PEG Droplets

**Figure 2:**
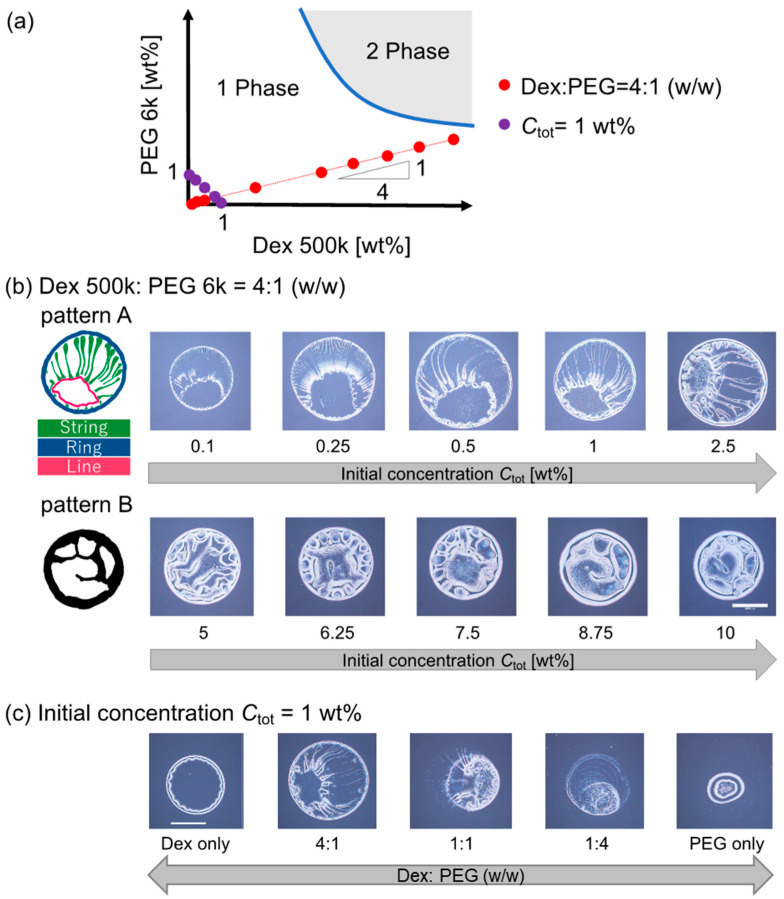
Evaporation patterns for binary polymer droplets of dextran 500 k and PEG 6 k. (**a**) Schematic of the dextran–PEG phase diagram. Red and violet points show the initial concentration and composition of the prepared droplet before the evaporation. The functions of the red and violet points are y = 0.24x and y = 1 − x, respectively. (**b**) Evaporation patterns of dextran and PEG droplets with different initial polymer concentrations *C*_tot_ under a fixed polymer ratio, dextran: PEG = 4:1 (*w*/*w*). The compositions are indicated with a red line in (**a**). (**c**) Evaporation patterns of dextran and PEG droplets with a different polymer ratio, under fixed *C*_tot_ = 1 wt%. The compositions are indicated with a violet line in (**a**). Scale bar: 1 mm.

We observed the evaporation patterns of binary polymer droplets containing dextran 500 k and PEG 6 k. Figure 2a shows a schematic phase diagram of the dextran 500 k and PEG 6 k solutions at room temperature. This dextran–PEG solution is known to exhibit phase separation upon condensation by evaporation. First, the dextran–PEG ratio was fixed to be 4:1 (*w*/*w*); the initial total polymer concentration *C*_tot_ was changed along the red line in Figure 2a. As shown in Figure 2b, the evaporation patterns of the dextran–PEG droplets show change with an increase in *C*_tot_. When the *C*_tot_ is less than 5 wt%, a ring pattern with multiple strings was observed (Figure 2b (top)). The multiple strings extend inward from the outer ring, as illustrated in Figure 2b (top left). Here, we refer to this narrow ring pattern with multiple strings as *pattern A*. As for higher *C*_tot_ ≥ 5 wt%, a thicker ring pattern than pattern A was observed (Figure 2c, bottom). Inside the thick ring, there are some structures, but not strings. To classify evaporation patterns by the presence or absence of internal strings, we refer to this thick ring pattern without strings as *pattern B*.

The ring-like structure common to patterns A and B might correspond to the coffee ring pattern upon evaporation. The coffee ring pattern represents a colloid deposition onto a droplet periphery due to colloid–laden droplet evaporation from the contact line, which creates the capillary flow that carries colloids to the contact line from the inside of the droplet [1]. In addition, an unclear internal structure of the pattern B for higher *C*_tot_ ≥ 5 wt% is quite similar to the one of previous reports using similar systems (dextran 10 k and PEG 8 k [13]). On the other hand, multiple strings of the pattern A for lower *C*_tot_ < 5 wt% is a novel pattern to the best of our knowledge. Hereafter, we focus on the pattern A with multiple strings inside.

To clarify the conditions under which the pattern A appears, we then fixed the initial polymer concentration *C*_tot_ = 1 wt% and changed the dextran–PEG ratio to be 1:0, 1:4, 1:1, 4:1, and 0:1 (*w*/*w*). The examined polymer compositions are indicated as violet points in the phase diagram (Figure 2a). As shown in Figure 2c, the pattern A (i.e., ring pattern with multiple strings) was again observed for the dextran–PEG with a ratio = 4:1 (*w*/*w*) as shown *C*_tot_ to 1 wt% in Figure 2c (*C*_tot_ < 5 wt%). The patterns for dextran: PEG = 4:1 and 1:1 look like pattern A; however, the strings extended from the outer ring were less obvious compared to the pattern for dextran: PEG = 1:4. 

**Figure 3:**
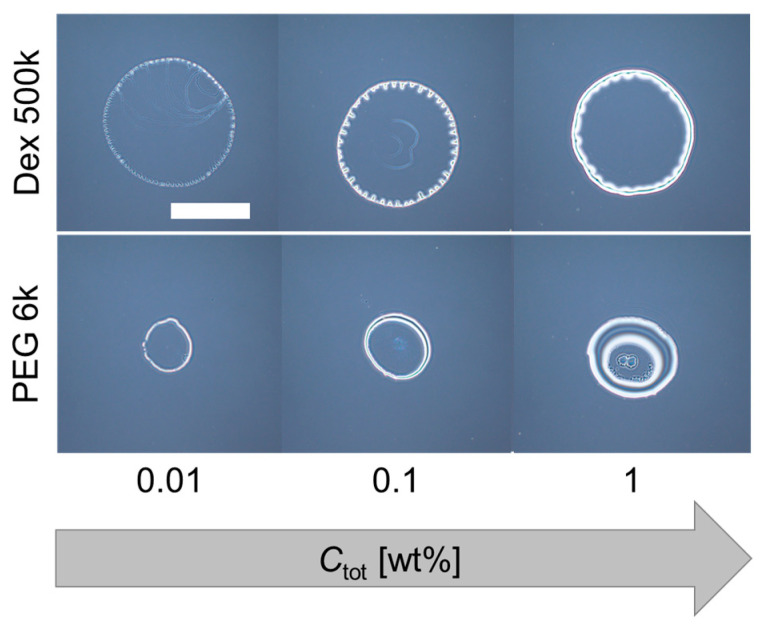
Deposition patterns of dextran only (**top**) and PEG only (**bottom**) droplets with different initial concentrations. Scale bar: 1 mm.

As for the PEG-only droplet (dextran: PEG = 0:1), a thick ring pattern without strings was observed for all *C*_tot_ (Figure 3 (bottom three images)). On the other hand, for the dextran-only droplet (dextran: PEG = 1:0), an undulated thick ring pattern was observed (Figure 3 (top three images)). This undulation at the contact line of the evaporating droplets was observed for dextran-only droplets, but not for PEG-only droplets. Therefore, the multiple periodic strings of the dextran–PEG droplets (pattern A) might have some relationship with the undulated pattern of the dextran-only droplets. To confirm the idea, we next replace the dextran with glucose which shows no phase separation and lower viscosity.

### 3.2. Evaporation Patterns for Glucose-PEG Droplets

**Figure 4:**
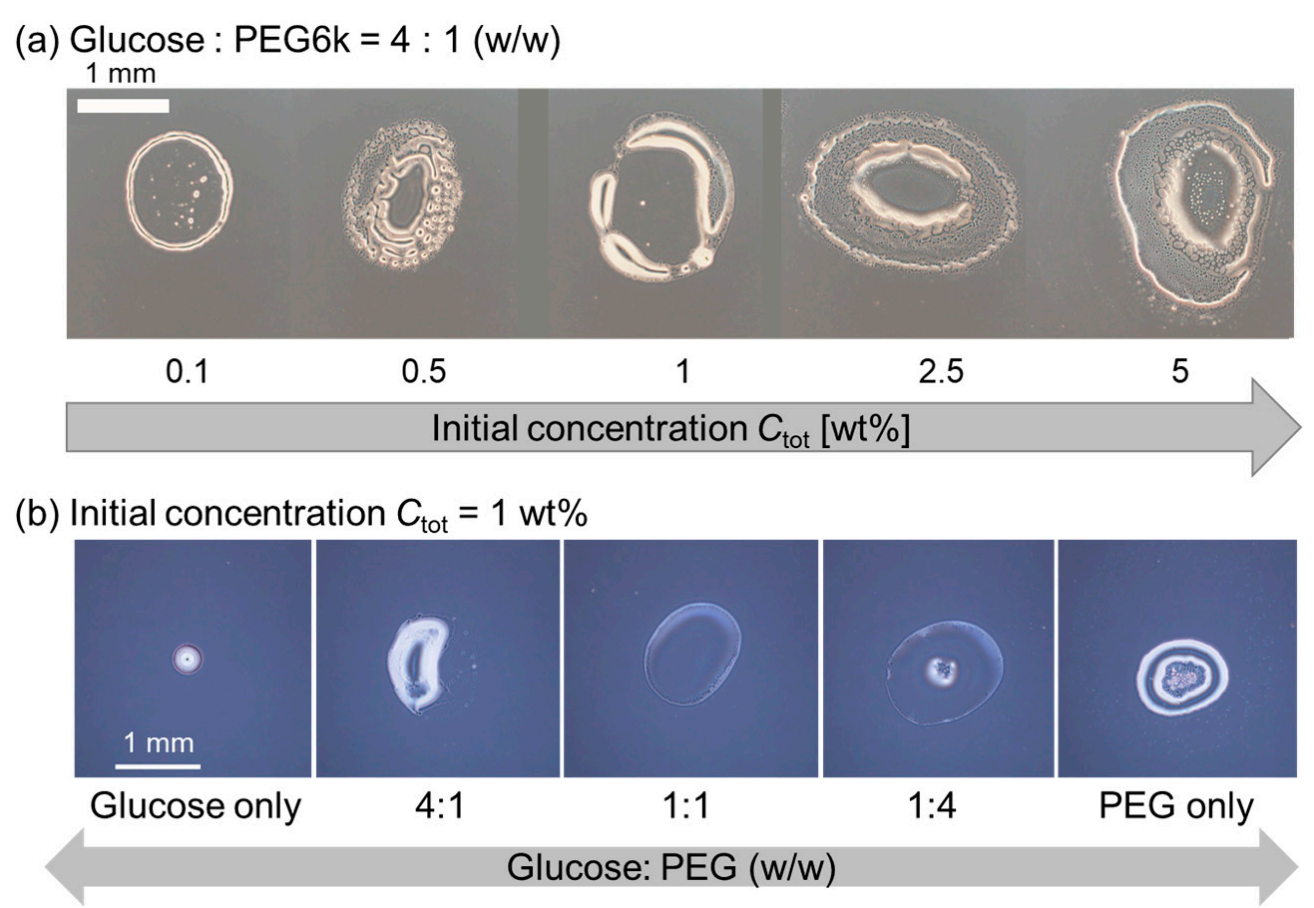
Evaporation patterns for droplets of glucose and PEG solutions. (**a**) Constant polymer ratio, glucose: PEG = 4:1 (*w*/*w*) with different initial polymer concentrations *C*_tot_. (**b**) Constant *C*_tot_ = 1 wt% with a different polymer ratio, glucose–PEG.

To verify whether dextran is the origin of the multiple strings in pattern A, dextran was replaced by glucose, the monomer unit of dextran (Figure 4). First, we changed the initial polymer concentration *C*_tot_ under a constant polymer ratio, i.e., glucose: PEG = 4:1 (*w*/*w*). For any *C*_tot_ examined, thick ring patterns without internal structure were observed (Figure 4a). Next, we fixed *C*_tot_ = 1 wt% which corresponds to the *C*_tot_ of the dextran and PEG system, showing pattern B with multiple stripes (Figure 2b, upper), and then varied the glucose–PEG ratio. Similarly, there were no string patterns; however, thick ring patterns without an internal structure or deposit on the center of the droplet were observed at any glucose–PEG ratio (Figure 4b). These results strongly support our idea that the coexisting condition of large dextran and PEG is a key to obtain the pattern A, i.e., multiple strings extend inward from the outer ring.

### 3.3. Fluctuation of Polymer Concentration in Evaporating Dextran–PEG Droplets

From these results, pattern A (ring pattern with multiple strings inside) is found to be characteristic evaporation pattern for dextran and PEG droplets with a low *C*_tot_ < 5 wt%. In addition, undulation of the dextran at the contact line seems to be related to the presence of multiple strings extended from the outer ring. To highlight the relationship between the dextran and string pattern, we visualize the concentration fluctuation upon the evaporation by using FITC-labeled dextran and RB-labeled PEG.

**Figure 5:**
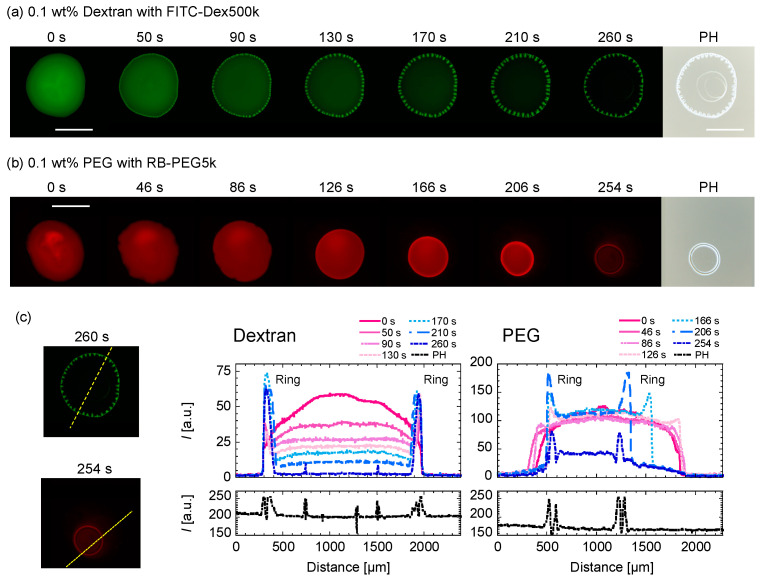
Time evolution of (**a**) C_tot_ = 0.1 wt% dextran-only and (**b**) C_tot_ = 0.1 wt% PEG-only droplets upon evaporation. The green and red represent (**a**) FITC–Dex and (**b**) RB-PEG, respectively. The phase contrast images on the far right (PH) are taken after ~265 s. Scale bar: 1mm. (**c**) Intensity line profiles of the dextran or PEG-only droplets at different times (**right** graphs). The intensity profiles were taken along the yellow dotted lines shown in the fluorescence images (**left**).

The evaporation processes for (a) dextran-only droplet with *C*_tot_ = 0.1 wt% and (b) PEG-only droplet with *C*_tot_ = 0.1 wt% are shown in Figure 5. For the dextran-only droplet, the ring pattern appears within 50 s (Figure 5a). The dextran-rich ring at the periphery of the droplet and transits to periodic domains upon the concentration fluctuation. From the phase contrast (PH) image taken after ~265 s, the second and third ring pattern inside are shown (Figure 5a (far right)). On the contrary, the PEG-only droplet recedes as time evolves, leaving a thick ring-like deposition without any internal structure (Figure 5b). The ring pattern appeared after ~166 s, which is slower than dextran. We can see that the ring becomes thicker as the PEG polymers at the droplet center move to the ring. In fact, there are no pattern inside the ring, as shown in the PH image (Figure 4b, right edge). This phenomenon is similar to that of the coffee ring pattern. To quantify the time development of the dextran concentration along the droplet periphery, we analyze the fluorescence intensity of FITC–dextran with time along the cross-sectional plane (dotted line in Figure 5c). Figure 5c (left) shows that the distances between the dextran-rich domains shortened over time, and the shape of the domains elongated toward the droplet center. On the other hand, the outer position of the dextran-rich domain did not change with time and was found to be almost at the same position after 260 s. This is in contrast to PEG in Figure 5c (right), where the outer position of the droplet moved significantly with time.

**Figure 6:**
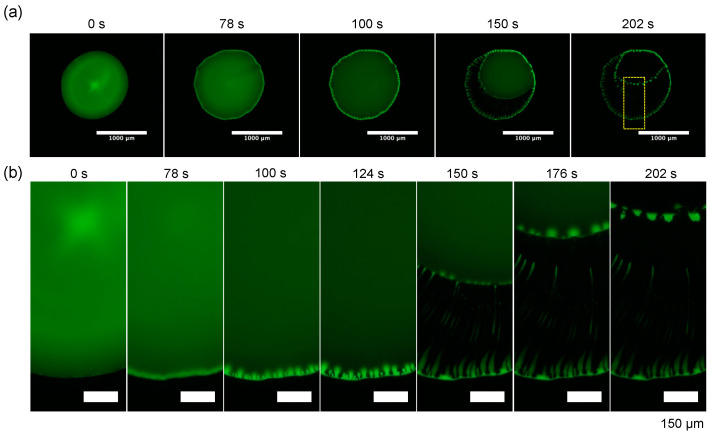
Time evolution of the evaporation patterns for dextran–PEG droplet with C_tot_ = 0.1 wt% and polymer ratio dextran: PEG = 4:1 (*w*/*w*). Fluorescence images of FITC–Dex (green). (**a**) Whole droplet images with a scale bar: 1 mm; (**b**) zoomed views of the part of the droplet surrounded by the yellow square (**a**). Scale bar: 150 µm.

**Figure 7:**
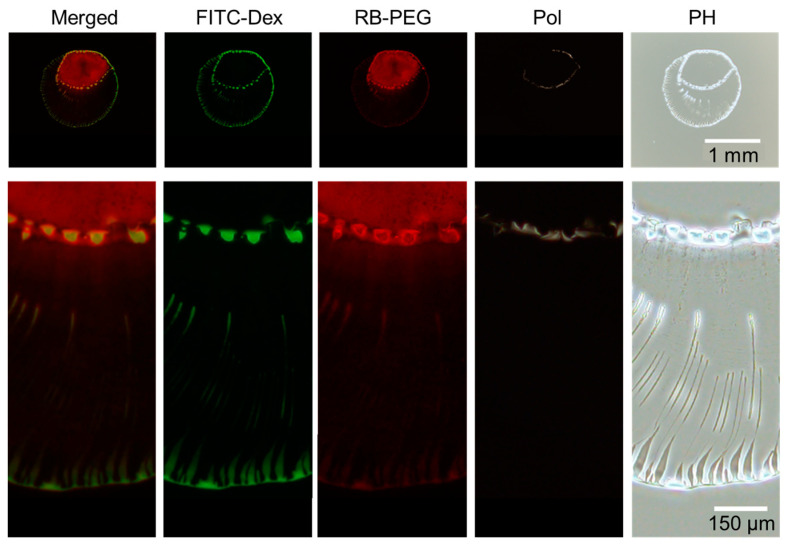
The final deposition images of the droplet shown in Figure 6). From left to right: Merged image of FITC–Dex (green) and RB-PEG (red), FITC–Dex, RB-PEG, simple polarized and phase-contrast microscopy images. Intensity modified for merged and RB-PEG images to obtain better visibility.

Finally, the time evolution of the evaporation pattern for dextran and the PEG droplet with *C*_tot_ = 0.1 wt% and the polymer ratio dextran: PEG = 4:1 (*w*/*w*) (Figure 6 and Figure 7). The whole droplet images and the zoomed images clearly show that dextran firstly condensates at the droplet periphery at 78 s and the dextran-rich ring pattern transits to periodic domains. This pattern transition is quite similar to the one of dextran-only droplet (Figure 5a). After 78 s, as the contact line of the PEG-rich phase receded, and the periodic dextran-rich domains were pulled toward the droplet center and transited into multiple strings. This formation mechanism of pattern A with a macroscopic flow from outer to inner is different from “viscous fingering” where a high viscosity fluid is extruded into a low viscosity fluid [16].

After 176 s, the dextran again accumulated to form an inner ring of multiple domains (Figure 6). The visualization of the PEG reveals that the PEG-rich locates inside the inner ring of dextran. The line dividing the inner dextran ring and PEG phase is found to be the phase boundary between dextran and PEG (Figure 7). A closer look at Figure 7 reveals that PEG-rich domains also exist just inside the ring of dextran domains, unlike PEG-only droplets (Figure 3). Therefore, the presence of dextran-rich domains at the phase boundary may contribute to the formation of the PEG-rich domains.

Phase contrast images demonstrate that the inner ring is composed of PEG, unlike the outer ring mainly composed of dextran. In addition, the accumulated PEG ring seems to be bright in polarized image, suggesting that the accumulated PEG ring contains ordered structure, i.e., crystal [17].

### 3.4. Initial Contact Angles of Dextran and PEG Droplet

**Figure 8:**
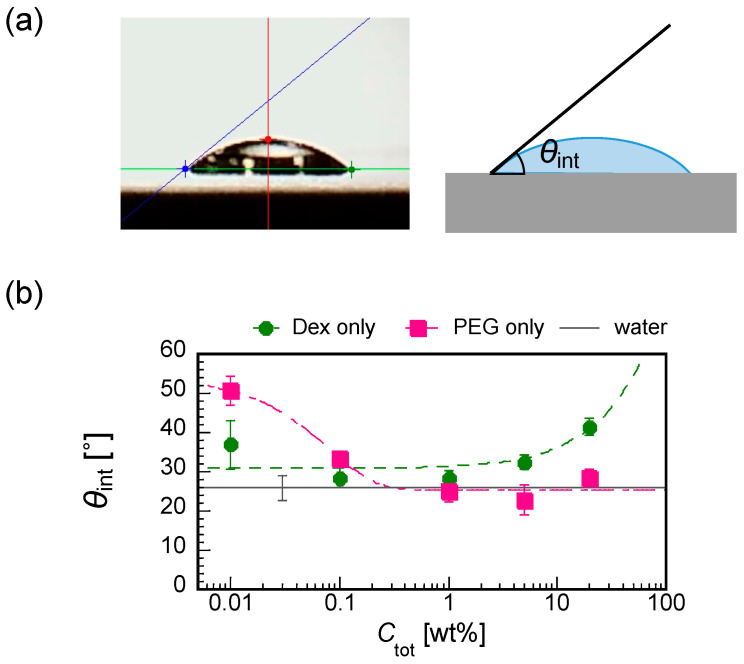
(**a**) Cross-sectional image of a droplet on a substrate showing how the contact angle θ_int_ is measured by the *θ*/2 method and the illustration. (**b**) The initial contact angles for different polymer concentrations for dextran-only (green closed circles) and PEG-only (red closed squares) droplets. The value for water of 25.9 ± 3.1(Ave. ± SD, *n* = 11) is shown with a solid black line with an error bar.

To understand the pattern A formation, we measured the initial contact angles θ_int_ for dextran-only and PEG-only droplets with some differnet *C*_tot_ from 0.01 to 20 wt% (Figure 8a). Interestingly, dextan and PEG showed the opposite trend of θ_int_ against *C*_tot_, i.e., the increase in *C*_tot_ increases the θ_int_ of the dextran droplet (lower wettability with increasing *C*_tot_ for dextran) and decreases the θ_int_ of PEG (higher wettability with increasing *C*_tot_ for PEG), comparable to the water (Figure 8b).

**Figure 9:**
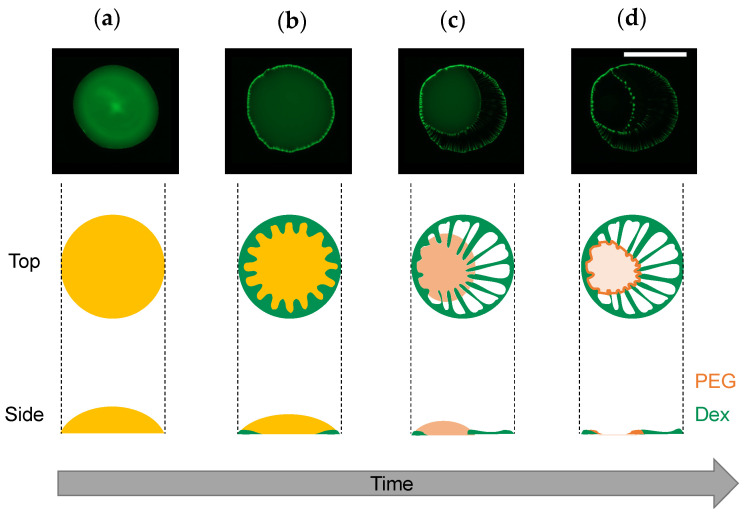
Illustrative time course of evaporation pattern formation. (**1**) Homogeneous solution just after the droplet deposition, (**2**) progression of phase separation at the contact line, (**3**) string formation by the PEG-rich phase receding to the center of the droplet, and (**4**) complete drying. The microscope images are the same as those shown in Figure 6 (image directions were changed for better visibility). Side views were assumed from the fluorescent intensity profiles. Scale bar: 1 mm.

Under the experimental conditions with *C*_tot_ = 0.01 to 1 wt% (Figure 3), the dextran and PEG have a differnet θ_int_ and a different relationship between *C*_tot_ and θ_int_. These differences can explain the heterogeneous distribution in the dextran–PEG droplet after 78 s, as shown in Figure 6. The dextran accumulated at the droplet contact line, but the PEG is rather central. At the initial evaporation stage with low *C*_tot_, the dextran with smaller θ_int_ than the PEG tends to wet the glass substrate. PEG solution easily recedes toward the center, which helps to ensure a stable large θ_int_. Upon evaporation, the θ_int_ of dextran-rich phase increases. It lowers the contact area of the dextran-rich phase on the substrate and helps to form elongated string-like patterns pulled by the PEG-rich flow. On the contrary, the θ_int_ of PEG-rich phase decreases upon evaporation. Initially, the contact line of the PEG solution is moved to maintain the large θ_int_; however, at some point, the contact line stops moving and wets the substrate. Since the PEG contact lines do not move, they cannot pull the dextran domains at the phase bundary and change them into strings. In fact, Figure 7 demonstrates that the dextran-rich domains at the phase boundary have a spherical shape unlike the elongated shape of dextran connected to the outer ring pattern. From the above, we conclude that the evaporation pattern A (ring pattern with multiple strings) originates from the difference in contact angles between PEG and dextran at a low concentration. In other words, upon evaporation, the dextran-rich phase with a small contact angle accumulates at the contact line of the droplet and forms multiple domains due to density fluctuation, and the PEG phase with large wetting angle deforms it into a string by pulling it. The illustrative time course of the pattern formation is shown in Figure 9.

## 4. Conclusions

We found a specific evaporation pattern of a dilute dextran–PEG droplets accompanied with phase separation. This pattern consists of multiple strings of dextran pulled from the initial contact line toward the center of the droplet and a concentrated circle of PEG. This pattern was not previously observed for a system using a higher polymer concentration and a lower molecular weight of dextran [13], nor dextran substituted to glucose, which is the monomer unit of dextran. Under our condition, while the macro-phase separation occurs at a rather late stage (due to the low initial concentration), the difference in substrate wettability between PEG and long dextran is large, even in the initial stage (as shown in Figure 8). These features may have altered the kinetic coupling between compatibility and wettability, resulting in the characteristic string pattern we found here. This pattern derives from the evaporation mechanism that starts from the very dilute region of the polymer blend, where changes in wettability to the substrate and also differences in compatibility between polymers occur simultaneously. The wettability changes of dextran and PEG to the substrate are opposite, i.e., decreasing for dextran and increasing for PEG. In addition, the decrease in their compatibility with time triggers phase separation. The novel pattern formation found in this study is attributed to the unique kinetic coupling between wettability and compatibility. The study of such complicated evaporation patterns of dilute polymer droplets can be important for diagnosing diseases using a small amount of blood and for considering the primitive environment in which life occurred. The key to understanding the observed phenomenon is the timing of macro-phase separation starts and the time development of the wettability. Therefore, in future work, we would like to analyze the local concentration change and the time evolution of the contact angle to the mixed solution. In addition, the latter can be controlled by the addition of surfactants as tried for the coffee-ring pattern and other studies [18,19]. Moreover, we would like to analyze the viscosity of the solution since it potentially affects the pattern formation.

## Figures and Tables

**Table 1:** Radius of gyration *R*_g_ and approximate overlap concentration c* of PEG 6 k and Dex 500 k estimated as same as [15].

	PEG 6 k	Dex 500 k
*R*_g_ [nm]	3.1	17
c* [wt%]	8	4

## Data Availability

Not applicable.
